# Fast food consumption and its associations with heart rate, blood pressure, cognitive function and quality of life. Pilot study

**DOI:** 10.1016/j.heliyon.2019.e01566

**Published:** 2019-05-17

**Authors:** Mohammad Alsabieh, Mohammad Alqahtani, Abdulaziz Altamimi, Abdullah Albasha, Alwaleed Alsulaiman, Abdullah Alkhamshi, Syed Shahid Habib, Shahid Bashir

**Affiliations:** a College of Medicine, King Saud University, Riyadh, Saudi Arabia; b Neuroscience Center, King Fahad Specialist Hospital Dammam, Dammam, Saudi Arabia

**Keywords:** Food science, Neuroscience

## Abstract

**Background:**

To investigate the relationship of fast food consumption with cognitive and metabolic function of adults (18–25 years old) in Riyadh, Kingdom of Saudi Arabia.

**Materials and Method:**

This cross-sectional study was conducted at the College of Medicine at King Khalid University Hospital, Riyadh, Saudi Arabia. The conventionally recruited subjects underwent an evaluation that included demographic data, quality of life (wellness, stress, sleepiness, and physical activity), mini-mental status examination, and the frequency of fast food consumption. To investigate metabolic function, blood was drawn to evaluate serum HDL, LDL, cholesterol, and triglyceride levels. Cognitive function was assessed by the Cambridge neuropsychological test automated battery. The participants were divided into 2 groups based on fast food consumption: those who consumed fast food 3 times per week or less (Group 1) and those who consumed fast food more than 3 times per week (Group 2).

**Results:**

The mean diastolic blood pressure in Group 1 and Group 2 was 72 mmHg and 77 mmHg, respectively, a significant difference (p = 0.04). There was no significant difference for cognitive function and quality of life between the two groups. There was significant correlation of HDL with AST correct mean latency and the AST correct mean latency congruent (p = 0.02, p = 0.01, respectively) and TC with diastolic blood pressure (p = 0.003).

**Conclusions:**

We concluded that fast food consumption has an effect on blood pressure but has no direct effect on cognition or quality of life.

## 1 Introduction

Fast food consumption has increased significantly worldwide. Fast food typically refers to food that is quickly prepared, rich in saturated fat, purchased from restaurants using precooked ingredients, and served in a packaged form [1]. Previous studies have shown that a high intake of sweetened beverages increases cardio-metabolic risk factors, obesity [2, 3, 4, 5], DM2 [6, 7, 8], hypertension [9], and metabolic syndrome [8, 10]. The rise in obesity rates in American adults (68.8%) currently classified as overweight or obese [11] link to increased intake of sugar-containing beverages [2]. The prevalence of DM2 in the Kingdom of Saudi Arabia is also increasing; among 6024 participants, diabetes mellitus was present in 1792 (30%) patients [12]. Studies have shown impaired cognitive functioning in DM2, and obesity [13]. Cognitive impairment in older age is neuropsychological marker of dementia [14, 15]. It is worth mentioning that fast food consumptions is increasing among Saudis in different age groups for both genders [16], as expected (Collison et al., 2010) found that the average fast food intake was 4.47 meal/week in different age groups, although girls consumed more fast food than boys [17]. Different populations based study showed different rate of food consumption/week [18, 19, 20, 21, 22, 23, 24].

Fast food has many unpleasant health consequences. It negatively affects brain health by damaging regions relevant to memory tasks and by diminishing brain-derived neurotrophic factor levels [25]. This amplifies the risk of developing dementia and Alzheimer's disease later in life [26]. A high intake of Western food, characterized by high levels of saturated fat, was associated with increased serum total cholesterol (TC) and low-density lipoprotein cholesterol (LDL-C), with an 8% increase in the likelihood of having sustained high LDL-C [27]. In combination with a sedentary lifestyle, an increased prevalence has been noted of chronic non-communicable diseases, such as diabetes, heart disease, and cancer, which are estimated to account for 78% of all deaths (WHO, 2014). Thus, this diet is detrimental to the health and will aggravate existing lifestyle diseases [26, 28]. The most common risk factor for developing coronary heart disease in Saudi patients is the consumption of a high-fat diet which contains high levels of LDL-C [29, 30].

Several cross-sectional studies have found significant associations between poor nutritional status and behavioral disturbances [31, 32, 33, 34, 35], worse cognitive status [31], and more impaired functioning in adult daily living activities [36, 37, 38, 39]. While these studies have demonstrated an association between nutritional status and aged population, very limited have examined the association of cognitive function with food addiction in young population and the majority of the studies have been conducted in clinical samples. Considering the lack of studies conducted in Saudi Arabia or in the Middle East regarding fast food consumption and its effects on cognitive function, we hypothesized that increased consumption of fast food would impair cognitive function, metabolic functions, and quality of life in young adults.

## 2 Materials & methods

This cross-sectional study was conducted in the Department of Physiology, King Khalid University Hospital (KKUH), Riyadh, Kingdom of Saudi Arabia. The conventionally recruited subjects underwent evaluation of demographic data, quality of life, mini-mental status examination, and the frequency of fast food consumption. Subjects also completed the Cambridge neuropsychological test automated battery (CANTAB).

### 2.1 Participants

A non-probability convenience sampling technique was used to recruit subjects. Our inclusion and exclusion criteria were designed to isolate the effect of fast food consumption and to exclude other possible causes of cognitive impairment. All subjects were healthy with no neurological or psychiatric disorders and were taking no medications. We included 60 healthy Saudi young adults ranging in age from 19 to 23 years old (mean age: 20.8 years). Judging by the average fast food consumption of young Saudi adults which is estimated to be 4.47 meal per weak we thought to categorize our subjects into two groups below and above average [17]. The sample was divided into two groups according to their fast food consumption. Group 1 included those who consumed fast food 3 times per week or less (n = 35; men = 30, women = 5), and the participants' mean age was 21.23 years. Group 2 included those who consumed fast food more than 3 times per week (n = 25; men = 21, women = 4). Demographic data are presented in Table 1. This study was conducted according to the guidelines of the Declaration of Helsinki, and all procedures were approved by the institutional review board of KKUH. Written informed consent was obtained from all participants.

**Table 1:** Demographic table for two groups.

	Group 1 n=35		Group 2 n=25	
	No	%	No	%
***Sex***				
*Male*	30	85.7%	21	84%
*Female*	5	4.3%	4	16%
***Family history of cognitive impairment***				
*yes*	4	11.42%	2	8%
*no*	31	88.58%	23	92%
***Medication***				
*yes*	3	8.57%	1	4%
*no*	32	91.43%	24	96%
***Physical activity***				
*none*	11	31.4%	12	48%
*some*	16	45.73%	6	24%
*regular*	8	22.87%	7	28%

### 2.2 Procedure and assessment

#### 2.2.1 Demographic

Data questionnaire was designed to assess the socioeconomic and lifestyle characteristics including smoking history, living situation, marital status, and medical history.

#### 2.2.2 Body composition

Body composition analysis was obtained via bioelectrical impedance analysis with a commercially available body analyzer (TANITA, USA). The subjects were asked to wipe the soles of their feet with a wet tissue and then to stand on the electrodes of the machine. Data was then recorded.

### 2.3 Cognitive function

Cognitive function was evaluated using the MMSE and CANTAB tests. The MMSE is one of the most widely used tools for quantitative assessment of cognitive function. The test consists of 11 questions assessing various cognitive functions, including 2 questions on orientation, 1 on registration, 1 on memory, 5 on language, 1 on attention and calculation, and 1 on visual construction. The test has a maximum score of 30, with scores below 23 being indicative of cognitive impairment.

#### 2.3.1 Delayed matching sample test

The delayed matching to sample simultaneously assesses visual matching ability and short-term visual recognition memory of patterns. The participant was shown a complex, abstract, visual pattern followed by four similar patterns after a brief delay. The participant selected the pattern that exactly matched the original pattern.

#### 2.3.2 Attention switch task (AST) test

The AST tests the participant's ability to switch attention between the location of the arrow on the screen and its direction. This test was designed to measure top-down cognitive control processes involving the prefrontal cortex. The test shows an arrow that can point to either the right or left side of the screen and may appear on either the right or left side of the screen. Some trials displayed congruent stimuli (e.g., an arrow on the left side of the screen pointing to the left), whereas other trials displayed incongruent stimuli that required a greater cognitive demand (e.g., an arrow on the left side of the screen pointing to the right). The detail description of the task can be assessed from the website (www.cantab.org).

#### 2.3.3 Intra-extra dimensional set shift test

The intra-extra dimensional set shift is a test of rule acquisition and reversal. It features visual discrimination and attentional set formation maintenance, shifting, and flexibility of attention. This test is sensitive to changes in the frontostriatal areas of the brain and is a computerized analog of the Wisconsin Card Sorting Test. Two artificial dimensions were used in the test: the detail description of the task can be assessed from the website (www.cantab.org).

### 2.4 Fast food consumption

Participants reported their fast food consumption in the month before the survey. They were asked, “In the past month, how many times did you buy food at a restaurant where food is ordered at a counter or at a drive-through window?” They could respond using 1 of 9 frequency categories: never or rarely; 1 time per month; 2–3 times per month; 1–2 times per week; 3–4 times per week; 5–6 times per week; 1 time per day; 2 times per day; or 3 or more times per day. They were also given a list of the most popular fast food restaurants and were asked if they had gone to any of these restaurants in the past month.

### 2.5 Blood samples

Venous blood samples were collected from all participants after an overnight fast and analyzed for fasting blood glucose, total cholesterol (TC), triglycerides (TG), high-density lipoprotein (HDL), and low-density lipoprotein (LDL) by using the enzymatic calorimetric method.

### 2.6 Quality of life

Quality of life was measured by the Ferrans and Power Quality of Life Index, which measures the quality of life in terms of satisfaction with life. The quality of life index are used to weight satisfaction responses so that the scores reflect satisfaction with the aspects of life that is valued by the individual. The quality of life index produces five scores (health and functioning, psychological/spiritual, social and economic, and family domains).

### 2.7 Statistical analyses

We calculated response time (ms), and numbers of percent correct trail in AST measurement. We measured DMS percent correct response, percent correct simultaneous, IED total error and IED stages completed between two groups. Independent samples t-tests were used for continuous variables, and the χ 2 test was used for categorical variables. All the statistical analyses were performed using 21.0 software (formerly SPSS Statistics Inc.). P < 0.05 was considered to be statistically significant. All data are expressed as means ±standard deviation (SD).

## 3 Results

### 3.1 Participant characteristics and fast food consumption

Group 1 included 35 participants with a mean age of 21.23 years, and group 2 included 25 participants with a mean age of 21 years. The demographic data are summarized in Table 1.

The Fast Food Consumption (FFC) was determined by responding to the question, “How often (times/week) did you eat a meal or snack in Western-style FF restaurants (e.g., McDonald's, KFC, Pizza Hut) in the past one month?” Each FFC pattern was categorized as yes/no, and times of FFC per week (0, 1–2, and ≥3 times). Nearly 58% of the participants consumed less than 3 regular fast food meals per week, and 42% consumed 3 or more meals of fast food per week. Men consumed regular soft drinks more frequently than did women. All of the subjects were high school graduates which is expected in this age group especially in an urban city like Riyadh. Individuals who did not consume regular fast food smoked less, had a smaller waist circumference and a lower body mass index (BMI), and had a lower TG and higher HDL-cholesterol levels compared with those who consumed regular fast food daily.

### 3.2 Cognition

#### 3.2.1 CANTAB

**Fig. 1:**
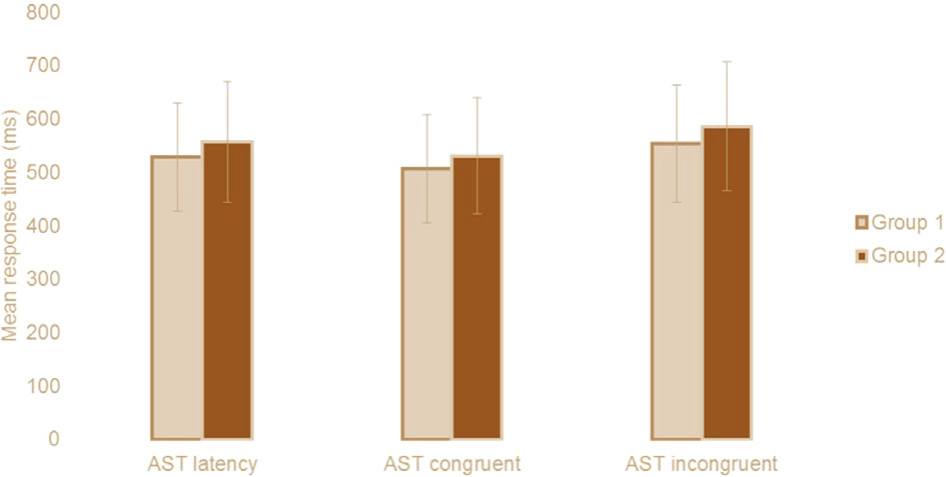
Mean response time (ms) for attention switching task correct latency, congruent and incongruent condition for two groups.

On the CANTAB test, There was not significant difference was found in the between two groups for AST congruency mean correct (Group 1 = 47.03 ± 57.34, Group 2 = 55.41 ± 41.17, t = −.625, P = 0.535, Table 2), AST Switching cost (Mean, correct) (Group 1 = 192.5 ± 133.8, Group 2 = 198.0 ± 140.6, t = −.156 P = 0.878, Table 2), AST Mean correct latency (Group 1 = 529.1 ± 101.4, Group 2 557.4 ± 113.2, t = −1.016, P = 0.314, Fig. 1; Table 2), AST mean correct latency (congruent) (Group 1 = 507.3 ± 101.2, Group 2 = 531.4 ± 109.4, t = −.876 P = 0.384, Fig. 1; Table 2), AST mean correct latency (incongruent) (Group 1 = 554.1 ± 109.6, Group 2 = 586.7 ± 120.2, t = −1.091 P = 0.280, Fig. 1; Table 2), AST mean correct latency (blocks 3,5) [non-switching blocks] (Group 1 = 437.7 ± 71.7, Group 2 = 462.0 ± 68.2, t = −1.322 P = 0.191, Table 2), AST mean correct latency (block 7) [switching block] (Group 1 = 624.9 ± 162.6, Group 2 = 660.1 ± 178.1, t = −.795 P = 0.430, Table 2) and AST percent correct trial (Group 1 = 92.7 ± 7.4, Group 2 = 92.4 ± 7.1, t = .191 P = 0.849, Table 2).

**Table 2:** Cambridge Neuropsychological Test Automated Battery (CANTAB) data for two groups.

	Group 1 n= 35	Group 2 n= 25	*P* value
	Mean ± SD	Mean ± SD	
AST Congruency cost	47.03 ± 57.34	55.41 ± 41.17	0.535
AST Switching cost	192.5 ± 133.8	198.0 ± 140.6	0.877
AST correct latency	529.1 ± 101.4	557.4 ± 113.2	0.314
AST correct latency (congruent)	507.3 ± 101.2	531.4 ± 109.4	0.389
AST correct latency (incongruent)	554.1 ± 109.6	586.7 ± 120.2	0.280
AST correct latency (blocks 3,5) [non-switching blocks]	437.7 ± 71.7	462.0 ± 68.2	0.191
AST correct latency (block 7) [switching block]	624.9 ± 162.6	660.1 ± 178.1	0.430
AST Percent correct trials	92.76 ± 7.44	92.40 ± 7.17	0.849
DMS Percent correct	90.42 ± 7.51	89.30 ± 5.70	0.530
DMS Percent correct (simultaneous)	96.57 ± 4.81	98.40 ± 3.74	0.118
DMS Percent correct (all delays)	88.38 ± 9.19	86.26 ± 7.14	0.346
IED Total errors (adjusted)	18.48 ± 15.88	17.92 ± 17.68	0.897
IED Stages completed	8.77 ± 0.64	8.68 ± 0.74	0.615

CANTAB test: CANTAB: Cambridge neuropsychological test automated battery, AST: Attention Switching Task, DMS: Delayed Matching to Sample, IED: Intra-Extra Dimensional Set Shift for two groups.

There was no significant difference for DMS percent correct response (t = .632, p = .530, Table 2) DMS percent correct simultaneous response (t = −1.586, p = .118, Table 2), DMS percent correct (t = .950, p = .436, Table 2), IED total error (t = .130, p = .897, Table 2) and IED stages completed (t = .506, p = .615, Table 2) between two groups.

### 3.3 MMSE

The MMSE score was reduced in Group 2 compared to Group 1, but this difference was not significant (t = −.186, p = 0.853) (Table 3).

**Table 3:** Blood pressure (systolic and diastolic, heart rate, MMSE and stress test for two groups.

	Group 1 n=35	Group 2 n=25	P value
	Mean ± SD	Mean ± SD	
***Blood pressur*e**			
*Systolic*	119.94 ± 9.65	122.56 ± 14.33	0.534
*Diastolic*	72.34 ± 9.46	77.28 ± 8.64	0.044
***Heart rate***	79.42 ± 13.34	81.68 ± 12.40	0.510
***MMSE***	28.47 ± 1.35	28.54 ± 1.47	0.853
***Stress Test***	3.76 ± 1.01	4.03 ± 124	0.360

MMSE: Mini Mental Status Examination.

### 3.4 Vital signs

Participants' blood pressure was measured by using a manual sphygmomanometer. The mean systolic blood pressure in the first group was 119 mmHg, and that in the second group was 122 mmHg (t = .626, p = .534, Table 3). The mean diastolic blood pressure in Group 1 and Group 2 was 72 mmHg and 77 mmHg, respectively, a significant difference (t = −2.063, p = 0.04, Table 3). The mean of heart rate in Group 1 was 79 beats per minute, and that in Group 2 was 81 beats per minute, a non-significant difference (t = −.663, p = 0.510, Table 3).

### 3.5 Body composition

There were no significant differences between Group 1 and Group 2, although body weight, BMI, fat percentage, and fat mass were higher in Group 2 (Table 4).

**Table 4:** Body composition analysis (height, weight, BMI, Fat%, Fat mass, TBW (kg) for two groups.

	Group 1 n=35	Group 2 n=25	*P* value
	Mean ± SD	Mean ± SD	
Height	172.4 ± 7.42	170.04 ± 8.73	0.26
Weight	74.38 ± 17.72	78.61 ± 26.48	0.46
BMI	24.92 ± 4.92	26.78 ± 8.03	0.27
Fat%	21.37 ± 7.79	24.65 ± 9.22	0.142
Fat mass	16.57 ± 8.90	21.06 ± 14.53	0.144
TBWkg	42.07 ± 8.19	42.14 ± 10.02	0.979

BMI: body mass index, TBW (kg): total body water.

### 3.6 Blood chemistry analysis

There was no correlation between fast food consumption and abnormal lipid panel findings between the two groups (LDL, t = 0.490, p = 0.626; HDL, t = 1.080, p = 0.285; TC, t = 1.085, p = 0.283; TG, t = −0.65, p = 0.949, Table 5).

**Table 5:** Blood chemistry analysis for two groups.

	Group 1 n=35	Group 2 n=25	*P* value
	Mean ± SD	Mean ± SD	
FBG	4.50 ± 0.84	4.54 ± 0.75	0.68
TC	4.32 ± 1.15	4.15 ± 0.92	0.283
TG	1.02 ± 0.50	1.05 ± 0.87	0.94
LDL	3.29 ± 0.98	3.29 ± 0.61	0.62
HDL	1.28 ± 0.37	1.23 ± 0.28	0.285

FBG: Fasting blood glucose, TC: Total cholesterol, TG: triglycerides, LDL: low-density lipoprotein, HDL: high-density lipoprotein for two groups.

### 3.7 Quality of life score

There was no statistically significant difference in the mean quality of life score between the two groups for all variable (Table 6).

**Table 6:** Quality of life questionnaire for two groups.

	Group 1 n=35	Group 2 n=25	*P* value
	Mean ± SD	Mean ± SD	
***Quality of life (1):***			
Total 1	137.48 ± 19.21	129.32 ± 30.07	0.20
Health 1	19.54 ± 0.73	19.60 ± 0.99	0.77
Social 1	19.81 ± 0.69	19.48 ± 0.87	0.10
Psyc 1	20.0 ± 0.92	19.74 ± 0.95	0.28
Family 1	20.13 ± 0.88	20.0 ± 1.04	0.59
***Quality of life (2):***			
Total 2	155.31 ± 11.93	158.04 ± 9.65	0.35
Health 2	20.44 ± 0.49	20.46 ± 0.43	0.83
Social 2	20.15 ± 0.62	20.15 ± 0.56	0.99
Psyc 2	20.73 ± 0.38	20.82 ± 0.25	0.13
Family 2	20.76 ± 0.52	20.84 ± 0.24	0.45

psyc: psychology.

### 3.8 Correlations

**Fig. 2:**
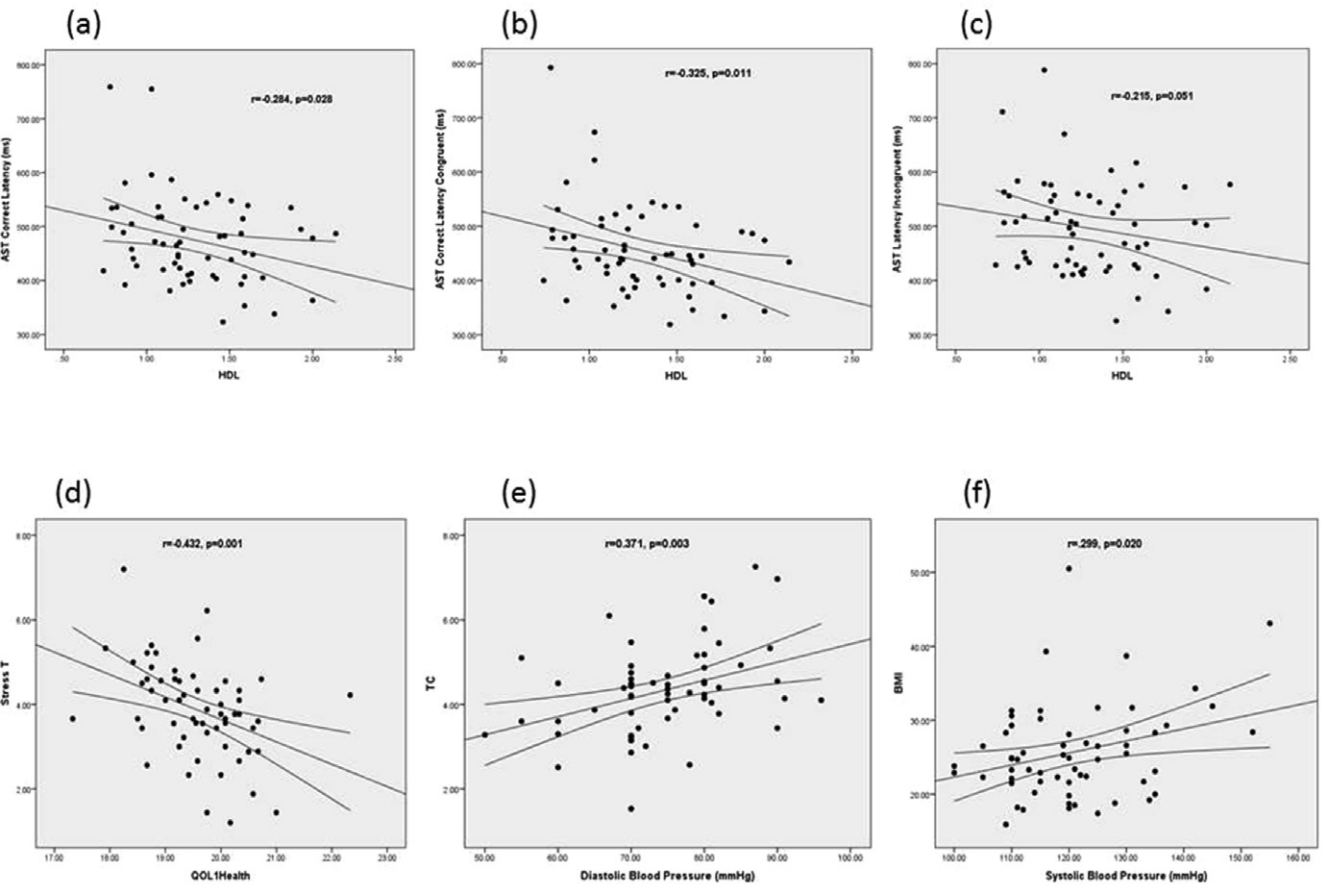
Correlation between HDL (high-density lipoprotein) and AST (cognitive attention switching) correct median latency (a).Correlation between HDL and AST correct median latency in congruent condition (b). Correlation between HDL and AST correct median latency in incongruent condition (c). Correlation between quality of life and stress (d). Correlation between diastolic blood pressure and TC (Total cholesterol) (e). Correlation between BMI (body mass index) and systolic blood pressure (f).

We found that increase in an HDL significantly decreased the AST correct mean latency, the AST correct mean latency congruent and AST correct mean latency incongruent (r = −0.284, p = 0.028, r = −0.325, p = 0.011, r = −0.215, p = 0.051 respectively) (Fig. 2a and b, c). The result showed that an increase in QOL 1 (health) was associated with a significant reduction in stress (r = −0.432, p = 0.001, Fig. 2d). An increase in diastolic blood pressure was significantly correlated with an increase in TC (r = 0.371, p = 0.003, Fig. 2e). This demonstrates that increased systolic blood pressure significantly correlated with an increase in BMI (r = 0.299, p = 0.020, Fig. 2f).

## 4 Discussion

The result showed significant effect of fast food on metabolic function but not cognitive function in healthy population.

One study from Ye et al [35] showed cognitive impairment (mostly memory function but other domains like attention and executive function were affected as well) in middle age group taking habitual sugar intake which included fruit drinks as well as soft drinks. In that study cognitive function assessed by the MMSE significantly correlated with sugars components like sucrose, glucose, and added fructose. It showed that an increase in the consumption of added sugar was significantly associated with lower MMSE scores. This differs from our study, in which fast food consumption was not associated with a number of specific cognitive domains, including attention, memory, working memory and executive function (Table 2). Our study might be different in terms of age group, fast food consumption and cognitive assessment measured tools [2, 3, 30]. The mechanism by which cognitive function is affected by diet is still not fully understood (Molteni et al., 2002), [25] found that HFS diet (high fat sucrose diet) was negatively associated with a decrease in hippocampal BDNF mRNA and protein, animals with higher BDNF had a better cognitive performance. Animals that were on HFS diet for a longer time exhibited lower levels of BDNF, which emphasizes on the importance of the duration of fast food consumption. The longer the duration of fast food consumption the lower the BDNF levels, although they found that 2 months on HFS diet were sufficient enough to reduce hippocampal levels of BDNF and spatial learning performance.

Our result showed metabolic difference in two groups which are in line of Raben et al. [38] study used to compare two group consume fast food for 10 weeks and increase blood pressure. The fructose in these beverages may stimulate an increase in TAG [39], [40]. High-fructose corn syrup play major role in obesity [41]. Fast food consumption on regular basis are major player for cardio-metabolic disease, including obesity, DM2, metabolic syndrome, and cardiovascular disease [5, 7, 8, 11] and all have negative impact on cognition [14, 15], [41]. Previous study lasting 6 months showed metabolic changes (visceral, liver, and muscle fat, TAG, TC, and systolic blood pressure) [42].

In KSA, over the past two decades, rapid economic development, global trade, and cultural exchange have meant that the FF industry and young population's FFC have been increasing rapidly. In this study, we defined Western FF as food sold in these fast food chains, e.g. KFC, McDonald's and Pizza Hut. This would make our estimate more conservative. So, we could not obtain information on the quantity of FFC, total daily energy intake, and FFC's contribution to total daily energy intake among the children. It may affect the assessment of the relationship between FFC and health outcomes.

This study has limitations which must be acknowledged. Our data is cross-sectional, and the dietary questionnaire used has a number of limitations. The sizes of fast food were not specified. Self-reported nutritional intake can lead to underestimation or overestimation of true associations, and measurement at only one point may not reflect long-term consumption patterns. Therefore, more studies, especially longitudinal studies based on large national representative samples with exact measures of quantity of fast food consume intake and its contribution to total daily energy intake, are needed to detect the association between fast food consume and health outcomes.

This is the first local cross-sectional study to examine fast food consumption and cognitive performance using a standardized battery of cognitive tests. It is very important in future to do longitudinal studies large, well-controlled, long-term interventional trials are needed locally.

## 5 Conclusion

In summary, the present study offers preliminary result for the effect of fast food consumption has an effect on metabolic function but has no direct effect on cognition or quality of life. More studies are warranted to understand.

For future research, we recommend that researchers should widen the study population and enroll a greater number of participants. In our study, we did not determine which cognitive domain was the most affected by fast food consumption, and thus, we encourage researchers to direct their research toward the most affected cognitive domain.

## Declarations

### Author contribution statement

Mohammad Alsabieh: Conceived and designed the experiments; Performed the experiments; Analyzed and interpreted the data; Wrote the paper.

Mohammad Alqahtani, Abdulaziz Altamimi: Conceived and designed the experiments; Performed the experiments; Wrote the paper.

Abdullah Albasha, Alwaleed Alsleman: Performed the experiments; Wrote the paper.

Abdullah Alkhamahi: Analyzed and interpreted the data; Wrote the paper.

Syed Shahid Habib: Contributed reagents, materials, analysis tools or data; Wrote the paper.

Shahid Bashir: Conceived and designed the experiments; Analyzed and interpreted the data; Contributed reagents, materials, analysis tools or data; Wrote the paper.

### Funding statement

This study was supported by a grant from Deanship of Scientific Research (Grant Number: RGP-1438-048) King Saud University, Riyadh, Saudi Arabia.

### Competing interest statement

The authors declare no conflict of interest.

### Additional information

No additional information is available for this paper.
